# A Text-Book of Chemical Physiology and Pathology

**Published:** 1892-10

**Authors:** 


A TEXT-BOOK OF CHEMICAL PHYSIOLOGY AND PATHOLOGY. By H. D. HALLIBURTON, M. D., B. Sc., M. R. C. P., Professor of Physiology at King's College, London; Lecturer on Physiology at the London School of Medicine for Women; late Assistant Professor of Physiology at University College, London. Illustrated. Pp. 874. London and New York: Longmans, Green & Co. 1891.

The physician who wishes to be in the vanguard of his profession must needs do a large amount of collateral reading upon subjects which do not treat directly of medicine and surgery. And, perhaps, no collăteral branch is gaining so much importance as that of chemistry in its specific relation to the physiology and pathology of the human economy. The medical profession is much indebted to Professor Halliburton for the volume before us. It is the best and most complete volume on chemical physiology and pathology which, as yet, has appeared.

The author divides this work into six parts. The first part, covering fifty-five pages, treats exclusively of the methods of research and analysis. While the author does not attempt to give the details of analytical chemistry as they are found in volumes devoted especially to that subject, still the outline given is clear, concise, and an excellent index for those who wish to do work in the field of chemical physiology.

Part II. treats of The Chemical Constituents of the Organism. Here not only the ultimate elemental composition of the body is considered, but also the simpler and more complex organic constituents. The author also gives the properties and tests for these organic compounds. The last chapter is devoted to a brief, though excellent review upon ptomaines and leucomaines.

The Tissues and Organs of the Body forms the subject-heading for Part III. This part is an excellent one, and deals clearly and concisely with the physiological and pathological chemistry of all the organs and tissues of the body.

In the chapter devoted to Blood in Disease, an excellent review of the chemistry of progressive pernicious anemia is given.

Part IV. takes up the subject of Alimentation, is well written, and full of valuable information.

The excretions form the subject of Part V. Several chapters are devoted to the urine, and the subject is concisely, though quite exhaustively, treated.

The last part is devoted to General Metabolism. It is an excellent exposition of the subject, and is so concisely written that it must be entirely and carefully read to be fully appreciated. We recommend this volume most heartily to the careful study of every member of the medical profession.

Messrs. Longmans, Green & Co. are entitled to considerable praise for the excellent typography, and the neat and tasteful manner in which the work has been issued. J. A. M.

